# Reviews

**Published:** 1881-05

**Authors:** 


### Reviews.

---

**Aphorisms in Fracture.** By R. O. Cowling, M. D., Professor of Surgery University of Louisville.

This little volume embodies the last words of its distinguished author, who died in Louisville a few days ago. It contains 131 aphorisms and a criticism of Dr. Hamilton's treatment of fractures, besides criticisms of "the aphorisms" of several distinguished surgeons. The little book is very readable and contains in a short scope a perfect treatise of fractures and their treatment.

---

**A Treatise on Diseases of the Joints.** By Richard Barwell, F. R. C. S. Senior Surgeon and Lecturer on Surgery, Charing Cross Hospital. Illustrated by numerous engravings on wood. Second edition, revised and much enlarged. New York: William Wood & Co. 1881.

William Wood & Co. have done a good work in publishing this excellent book in their library of standard medical authors; it is a present which the profession cannot sufficiently appreciate. The first edition appeared many years ago, but this edition is so much enlarged, and contains in reality so little of the first edition, that it may be considered a perfectly new work. The pathology of the different joint affections is clearly and distinctly given: the symptomatology is exhaustive with many interesting reports of clinical cases added; the treatment is sensible and based upon scientific reasons. Altogether the volume is one of the most useful and interesting in the series of standard authors.

**Photographic Illustrations of Cutaneous Syphilis.** By GEORGE HENRY FOX, A. M., M. D., Clinical lecturer on the diseases of the skin, College Physicians and Surgeons, New York, etc , etc Forty-eight plates from life, colored by hand. New York : E. B. Treat & Co., 757 Broadway.

We have received Nos. 7, 8 and 9, of these admirable illustrations of the syphiloderma, a work for which our endorsement has been given in previous numbers of the journal.

No. 7 treats of syph. tuberculosum, syph. tuberculosum ulcerativum, syph. tuberculosum squamosum, syph. tuberculosum crustaceum.

No. 8 treats of syph. tuberculosum serpiginosum, syph. tuberculosum ulcerativum, scrofuloderma, syph. pustulo-crustaceum.

No. 9 treats of syphiloderma gumeatosum. Four cases.

The text is very clear in its description of the case illustrated in the plates. Prof. Fox is more than fulfilling the assurances given when undertaking the important work, and is conferring upon the profession an invaluable aid in the diagnosis and treatment of this important class of disease. We commend the work not only for the highly artistic execution of the illustrative plates, but for the practical character of the contents. We are gratified also to announce that it is received with increasing favor in this section where it has been introduced.

---

**Hobbs' Botanical Hand-Book** of Common Local English, Botanical and Pharmacopœial names arranged in alphabetical order, of most of the Crude Vegetable Drugs, etc., in common use; their properties, productions and uses, in an abbreviated form. Published by A. C. HOBBS, Somerville, Mass. Price, $2.

This work forms a complete index to the medical botany of nearly every drug in common use. The confusion which prevails among the common names of our native plants renders such a work as this most valuable to every physician and really indispensable to druggists. Our pharmaceutical friends who, without such a guide as this, are perplexed everyday through the multitude and vanity of the names borne by herbs, etc., will thank us for directing them to this excellent book.

**Drugs that Enslave—The Opium, Morphine, Chloral and Hashish Habits.** By H. H. Kane, M. D., New York. Philadelphia: Presley Blackiston. 1881.

Those who have read the excellent work of Dr. Kane, upon "The Hypodermic Infection of Morphine" will expect that this work will be well worth the buying. We can assure them against disappointment. The book contains a great deal of interesting material of a scientific and practical nature, based upon a thorough knowledge of the literature of the subject, and letters of nearly a thousand correspondents in various parts of the world. The abuse of narcotics is a growing evil to which the facts presented in this book should arouse attention.

---

**Atlas of Human Anatomy.** Containing 180 large plates, arranged according to Drs. Oesterreicher and Erdl, from their original designs from nature, and those of the greatest anatomists of modern times, viz: Weisse, Kiernau, Cloquet, Stilling, Blumenbach, &c., with full explanatory texts by J. A. JEANCON, M. D. Complete in 45 parts, at 75 cents each. A. E. Wilde & Co., publishers, Cincinnati, Ohio.

We directed attention to the initial numbers of this atlas in our January issue, since which time we have received Nos. xi to xxx inclusive. The assurances given in the prospectus have been fully carried out in the work, as far as we have been able to examine it. The size of each plate is sufficient to illustrate clearly the minute structure of every part. The work commends itself not only on account of the accuracy with which the human body is portrayed, but also for its artistic finish. The low price for which it is offered brings the work within the reach of the younger members of the profession whose knowledge of anatomy is fresh, and whose interest in this department is more active than in subsequent years, when the daily demands of their profession direct their minds to pathological and therapeutical studies, &c. The commendations heretofore given have been well merited, and we trust the enterprise of the publishers will be amply rewarded by the favor with which the work is received by the profession.

